# Supplementary material for: The PKG Inhibitor CN238 Affords Functional Protection of Photoreceptors and Ganglion Cells against Retinal Degeneration
Source: Int J Mol Sci. 2023 Oct 17;24(20):15277. doi: 10.3390/ijms242015277 (PMC10607377; doi:10.3390/ijms242015277)
Supplement: Supplementary file 1 [file ijms-24-15277-s001.zip › ijms-2541364-supplementary.pdf]

The PKG Inhibitor CN238 Affords Functional Protection of Photoreceptors and Ganglion Cells against Retinal Degeneration

Arianna Tolone [1,†], Wadood Haq [2,†], Alexandra Fachinger [3,‡], Akanksha Roy [4,‡], Sandeep Kesh [5], Andreas Rentsch [6], Sophie Wucherpfennig [5], Yu Zhu [1], John Groten [4], Frank Schwede [6], Tushar Tomar [4], Friedrich W. Herberg [3],
Vasilica Nache [5] and François Paquet-Durand [1,*]

[1] Cell Death Mechanism Group, Institute for Ophthalmic Research, Eberhard-Karls-Universität Tübingen, 72076 Tübingen, Germany; arianna.tolone@live.it (A.T.); yu.zhu@uni-tuebingen.de (Y.Z.)
[2] Neuroretinal Electrophysiology and Imaging, Institute for Ophthalmic Research, Eberhard-Karls-Universität Tübingen, 72076 Tübingen, Germany; wadood.haq@uni-tuebingen.de
[3] Biochemistry Department, University of Kassel, 34132 Kassel, Germany; alexfachinger@uni-kassel.de (A.F.); herberg@uni-kassel.de (F.W.H.)
[4] PamGene International B.V., 5211 ‘s-Hertogenbosch, The Netherlands; akanksha.roy.in@gmail.com (A.R.); jgroten@pamgene.com (J.G.); ttomar@pamgene.com (T.T.)
[5] Institute of Physiology II, University Hospital Jena, Friedrich Schiller University Jena, 07743 Jena, Germany; sandeep.kesh@med.uni-jena.de (S.K.); sop.wucherpfennig@yahoo.de (S.W.); vasilica.nache@med.uni-jena.de (V.N.)
[6] Biolog Life Science Institute GmbH & Co. KG, 28199 Bremen, Germany; ar@biolog.de (A.R.); fs@biolog.de (F.S.)
* Correspondence: francois.paquet-durand@klinikum.uni-tuebingen.de
† These authors should be considered as equal 1st authors.
‡ These authors should be considered as equal 2nd authors.

Statistics

1) PKG activation/inhibition assay: Data were analysed using GraphPad Prism 8.0.1 (GraphPad Software, Inc, San Diego, CA, USA). Activation (K_{act}) and inhibition (IC50) data are presented as mean ± standard deviation (SD); n = 3.

2) Heterologously-expressed CNG- and Kv1.3- channels and electrophysiology on *Xenopus laevis* oocytes: Data were analysed with the OriginPro 2016G software (OriginLab Corporation, Northampton, USA) using the two-tailed unpaired Student t-test (Table S2). Experimental values are given as mean ± SEM. Figures were prepared using CorelDraw® 2019 (Corel, Ottawa, Canada).

3) Retinal cell death: The number of TUNEL positive cells in the outer nuclear layer (ONL) was counted manually on pictures captured on at least six different, non-overlapping retinal areas, for at least four different animals for each genotype. Since one image at 20x magnification displayed a retinal circumference of just under 600 µm, the total retinal circumference covered by the analysis was approx. 3500 µm. Percentages of TUNEL positive cells were calculated by dividing ONL area by the average ONL cell size. Values obtained are given as fraction of total ONL cell number and expressed as mean ± SD.

4) Ganglion cell and cone survival: For retinal ganglion cell quantification, an area of 1 mm^2 was divided by the product of the length of counting and the section´s thickness (12 µm). RBPMS positive cells were counted manually. To count the surviving cones, the number of cone-arrestin-positive cells was divided by the ratio of 100 µm length to count length. For statistical analysis in both 3) and 4) a one-way ANOVA testing followed by Dunnett´s multiple comparison test as implemented in Prism 8 for Windows (GraphPad Software) was used.

5) Functional data: (A) MEA. To quantify PKG-inhibitor effects on retinal light sensitivity, photoreceptor (µERG) and ganglion cell (spikes) responses were considered: (1) Light responsiveness was represented as the percentage of electrodes detecting light-dependent µERG activity, to estimate retinal light-sensitivity and to indirectly infer the density of functional photoreceptors in each electrode recording field. Note that a single MEA electrode captured the integrated signal of multiple photoreceptors within the recording field. The 59 MEA electrodes with 40 µm spacing together span an overall recording field of 340 X 280 µm. A µERG response upon light stimulation was counted as light-responsive when the response amplitude exceeded the average of 500 ms control pre-stimulus baseline by 1.75-fold or more. (2) The µERG negative wave deflection reflects the strength of the photoreceptor light-evoked response - its hyperpolarization, equivalent to the a-wave in a conventional ERG - indicated by the initial negative µERG deflection (Figure 3 C, arrow). (3) Spike responses were accounted as light-stimulus correlated, if the post-stimulus activity (average of 6 bin counts: 500 ms light duration and 100 ms post-stimulation, 100

ms binning) exceeded the pre-stimulus activity (threshold: average of 5 bin counts pre-stimulus; 500 ms and 100 ms binning). Activity maps reflect the µERG recordings in their spatial context. Each pixel corresponds to one MEA recording electrode (centre to centre) and its surrounding recording area. The colour encodes the negative deflection of the recorded µERG (-5 µV binning). For statistical analysis of *rd10* data, one-way ANOVA followed by Dunnett´s multiple comparison test was used, while Wilcoxon-Mann-Whitney test was utilized for WT (MATLAB, The MathWorks, Natick, USA).

(B) Ca^{2+}-imaging. Ganglion cell traces were extracted from Ca^{2+}-imaging recordings (200 per recording) by encircling ganglion cells as region of interest (ROI, ~ 10-pixel diameter). The ROIs were drawn in ImageJ and the extracted traces were analysed using Matlab (MATLAB). For statistical analysis one-way ANOVA was applied, followed by Dunnett´s multiple comparison test (MATLAB).

6) STK data: Microarray images taken at multiple exposure times were combined to a single value in BioNavigator® and log2 transformed. Differences in phosphorylation signal intensities (significant differences $p < 0.05$) between *rd10* treated and untreated retinal samples for each peptide (KCNA1_438_450, KCNA2_442_454, KCNA3_461_473, KCNA6_504_516) were determined by unpaired t-test and visualized as Bar Plots (GraphPad Prism version 9.2.0).

Supplemental figures and tables

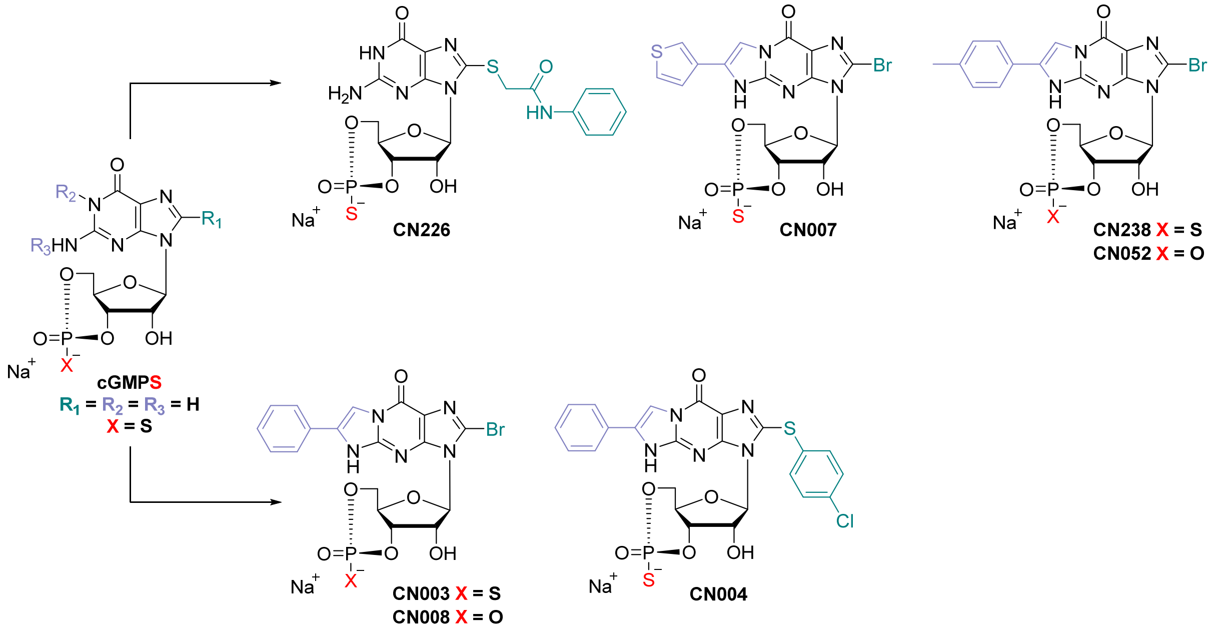

Figure S1. Structures of reference compounds and novel cyclic nucleotide analogues of cGMP. CN003 (Rp-8-Br-PET-cGMPS) and **CN004** (Rp-8-pCPT-PET-cGMPS) featured a cGMPS backbone containing a sulphur-modified phosphate in Rp-configuration, conferring PKG inhibitory properties (17). Further modifications were introduced on the nucleobase moiety at positions 8 (R_1) and 1, N^2 (R_2, R_3). While both reference compounds contain a so-called PET-group (β- **p**henyl- 1, N^2- **et**heno) at 1, N^2, this group is either lacking (**CN226** (Rp-8-PAmdMT-cGMPS)), substituted with an additional methyl-group (**CN238** (Rp-8-Br-pMe-PET-cGMPS)), or replaced through the heteroaromatic furan ring (**CN007** (Rp-8-Br-(3-Tp)ET-cGMPS)). At position 8 the residue in **CN226** contains a phenyl ring as present in reference compound **CN004**, however, a different, slightly larger linker has been introduced and said phenyl ring is unsubstituted. **CN007** and **CN238**, in turn, share the same bromide function as in the reference compound **CN003**. Compounds **CN008** (8-Br-PET-cGMP) and **CN052** (8-Br-pMe-PET-cGMP) were selected as the corresponding PKG activatory derivatives of **CN003** and **CN238** displaying a regular phosphate group instead of the sulphur modification. A compound concentration of 50 µM was used, based on previous *in vitro* results obtained with the reference compounds (16).

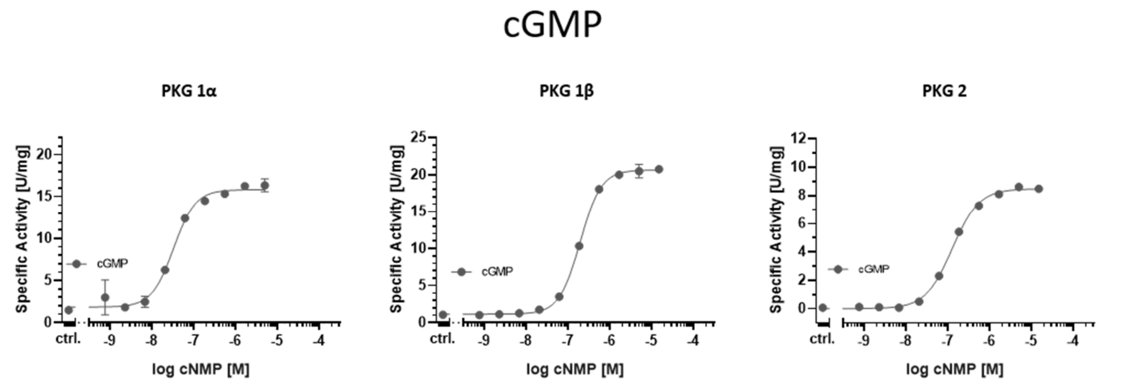

k_{act} = 28.1 ± 3.4 nM *(3)*	k_{act} = 155 ± 13 nM *(3)*	k_{act} = 140 ± 13 nM *(3)*

Figure S2. cGMP-dependent activation of the three distinct PKG isoforms. Activation curves of PKG1α, PKG1β, and PKG2 were obtained in three independent measurements with a cGMP dilution series ranging from 5 µM to 25.4 pm for PKG1α and from 15 µM to 762 pm for PKG1β and PKG2.

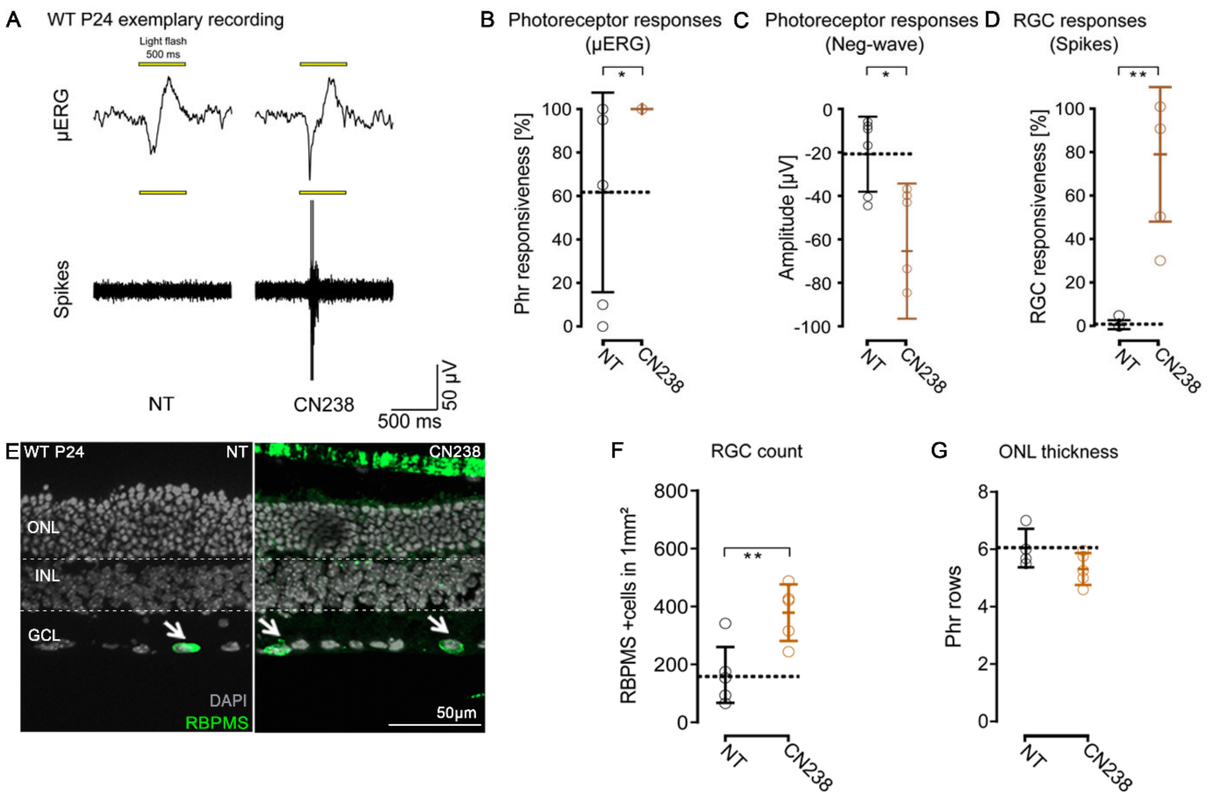

Figure S3. CN238 improves RGC viability and function in wild-type retinal explant cultures. (**A**) Representative micro-electroretinogram (µERG) recording (top) and light-correlated retinal ganglion cell (RGC) spike recordings (bottom) obtained from wild-type (WT) P24 retinal explants. Explants were treated from P14 to P24 with 50 µM CN238 and compared to non-treated (NT). (**B-D**) Quantification of light-stimulus evoked retinal activity in NT and treated WT explants: (**B**) light responsiveness indicated as % micro electrode array (MEA) electrodes detecting light-correlated µERG activity. (**C**) Quantification of negative µERG amplitudes. (**D**) RGC activity expressed as percentage of MEA electrodes detecting light-correlated spike-activity. (**E**) Sections derived from recorded WT P24 retinal explant cultures stained with DAPI (grey) and the RGC marker RBPMS (green). (**F**) Quantification of RBPMS positive cells in WT P24 retinal explant sections NT or treated with CN238. (**G**) Quantification of photoreceptor rows in NT and CN238 treated WT explants. Error bars indicate SD; statistical analysis: unpaired Student´s *t*-test; levels of significance: **P* ≤ 0.05, ***P* ≤ 0.01, ****P* ≤ 0.001. ONL = outer nuclear layer, INL = inner nuclear layer, GCL = ganglion cell layer.

A

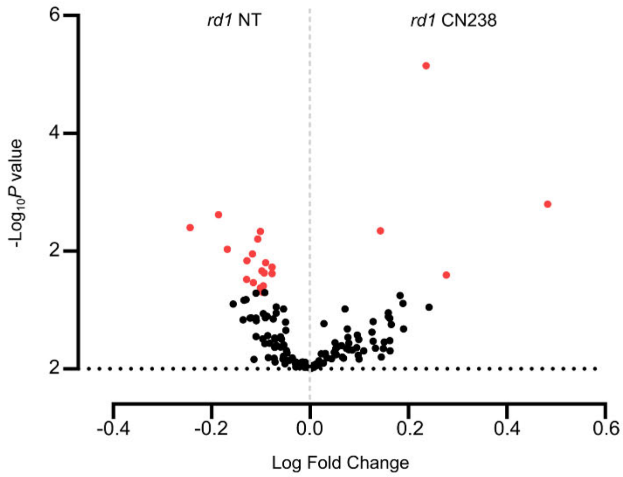

B

Rank	Peptide Name	UniProt ID	*p* Value	Log Fold Change
1	RAP1B_172_184	P61224	0.000	0.236
2	H2B1B_ 27_40	P33778	0.002	0.483
3	NEK2_172_184	P51955	0.002	-0.186
4	CA2D1_494_506	P54289	0.004	-0.244
5	GPR6_349_361	P46095	0.005	0.143
6	STK6_283_295	O14965	0.005	-0.101
7	GBRB2_427_439	P47870	0.006	-0.106
8	NCF1_296_308	P14598	0.009	-0.168
**9**	**KCNA6_504_516**	**P17658**	**0.011**	**-0.117**
10	AKT1_301_313	P31749	0.014	-0.128
11	CFTR_761_773	P13569	0.016	-0.090
12	F263_454_466	Q16875	0.019	-0.077
13	VTNC_390_402	P04004	0.022	-0.098
14	GRIK2_708_720	Q13002	0.023	-0.093
15	TOP2A_1463_1475	P11388	0.024	-0.077
16	RB_774_786	P06400	0.026	0.277
17	ESR1_160_172	P03372	0.030	-0.129
18	C1R_201_213	P00736	0.034	-0.115
19	CREB1_126_138	P16220	0.039	-0.095
20	PP2AB_297_309	P62714	0.042	-0.101
21	E1A_ADE05_212_224	P03255	0.046	-0.098

Figure S4. Phosphorylation status of Kv1.6 in the *rd10* mouse retina at P24. A) Volcano plot representing Log Fold Change (LFC) and −Log_{10} *p*-value for peptide phosphorylation. Red dots indicate significantly changed phosphopeptides (*p*-value < 0.05), black dots represent peptides with no significant alteration in phosphorylation. **B**) List of peptides whose phosphorylation significantly changed ($p < 0.05$) after CN238 treatment in *rd10* retinal explants with peptide name, UniProt ID, *p*-value, and log fold change. Note the decreased phosphorylation of KV1.6, here identified by its gene designation KCNA6.

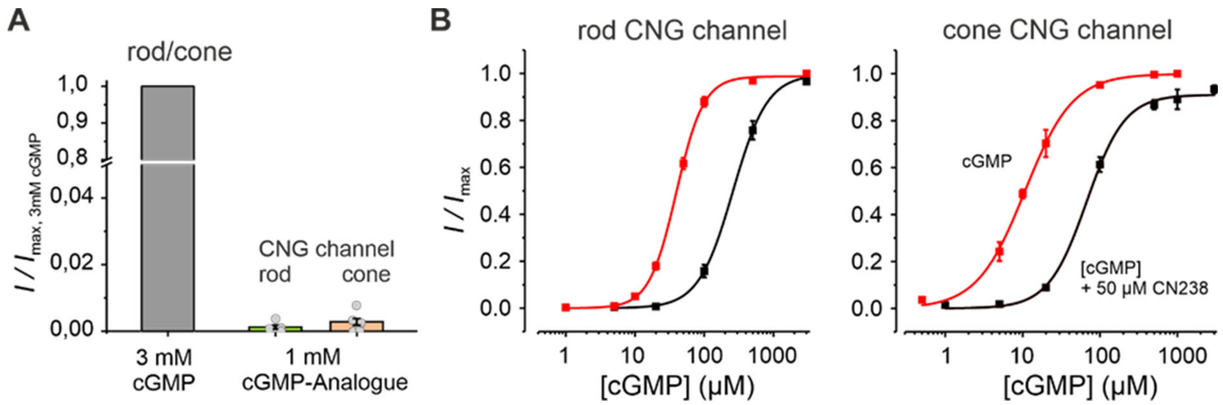

Figure S5. Effect of CN238 on retinal CNG-channels. A) Efficacy of CN238 when activating rod and cone CNG-channels. The currents triggered by 1 mM cGMP analogue were related to the saturating current induced by 3 mM cGMP (n = 5). The grey symbols represent individual measurements. No statistical difference was found between the results obtained with the two CNG-channel isoforms. **B**) Concentration-activation relationships for rod (left) and cone (right) CNG-channels in the presence of either cGMP (red) or cGMP + 50 µM CN238 (black). Curves were obtained by fitting the respective data points with Eq. 2 (n = 5-9 for rod; n = 5-13 for cone). In the presence of cGMP only, fitting yielded the following values: EC_{50} = 10.7 µM, H = 1.4 for cones and EC_{50} = 39.5 µM, H = 2.1 for rods. In the presence of cGMP and CN238 the values obtained were EC_{50} = 67.2 µM, H = 1.8 for cone and EC_{50} = 259.3 µM, H = 1.7 for rods.

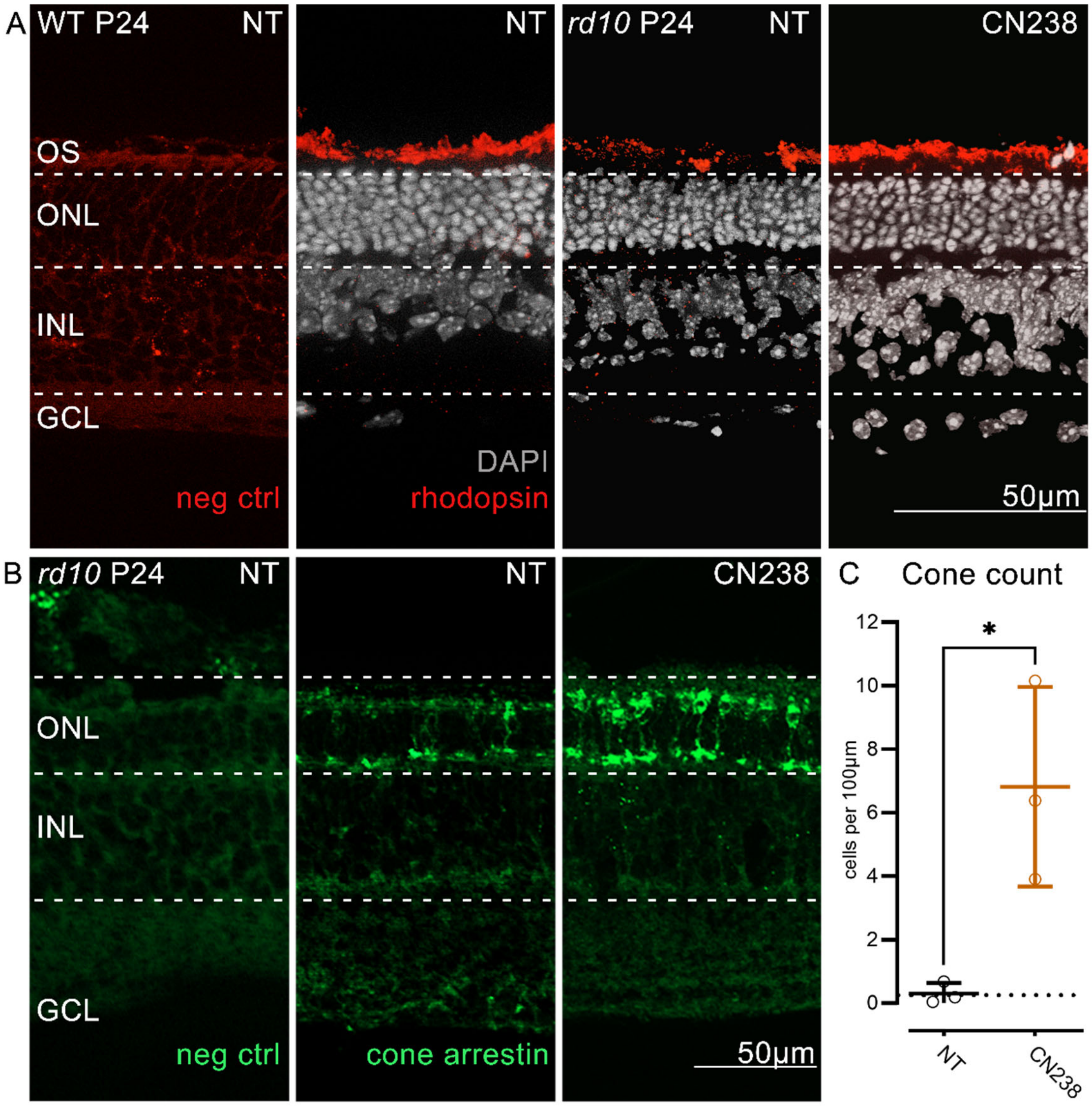

Figure S6. Rhodopsin and cone-arrestin staining in P24 retinal explant cultures. (**A**) Sections derived from non-treated (NT) and CN238-treated WT and *rd10* P24 retinal explant cultures stained with DAPI (grey) and rhodopsin (red). Labelling of rod outer segments appears more prominent in CN238 treated retina. (**B**) Sections derived from NT and CN238-treated *rd10* P24 retinal explant cultures stained with DAPI (grey) and cone-arrestin (green). (**C**) Quantification of cone-arrestin positive cells in *rd10* P24 retinal explants NT or treated with CN238. Note the marked improvement of cone survival afforded by CN238 treatment. Error bars indicate SD; statistical analysis: unpaired Student´s *t*-test; levels of significance: **P* ≤ 0.05, ***P* ≤ 0.01, ****P* ≤ 0.001. ONL = outer nuclear layer, INL = inner nuclear layer, GCL = ganglion cell layer.

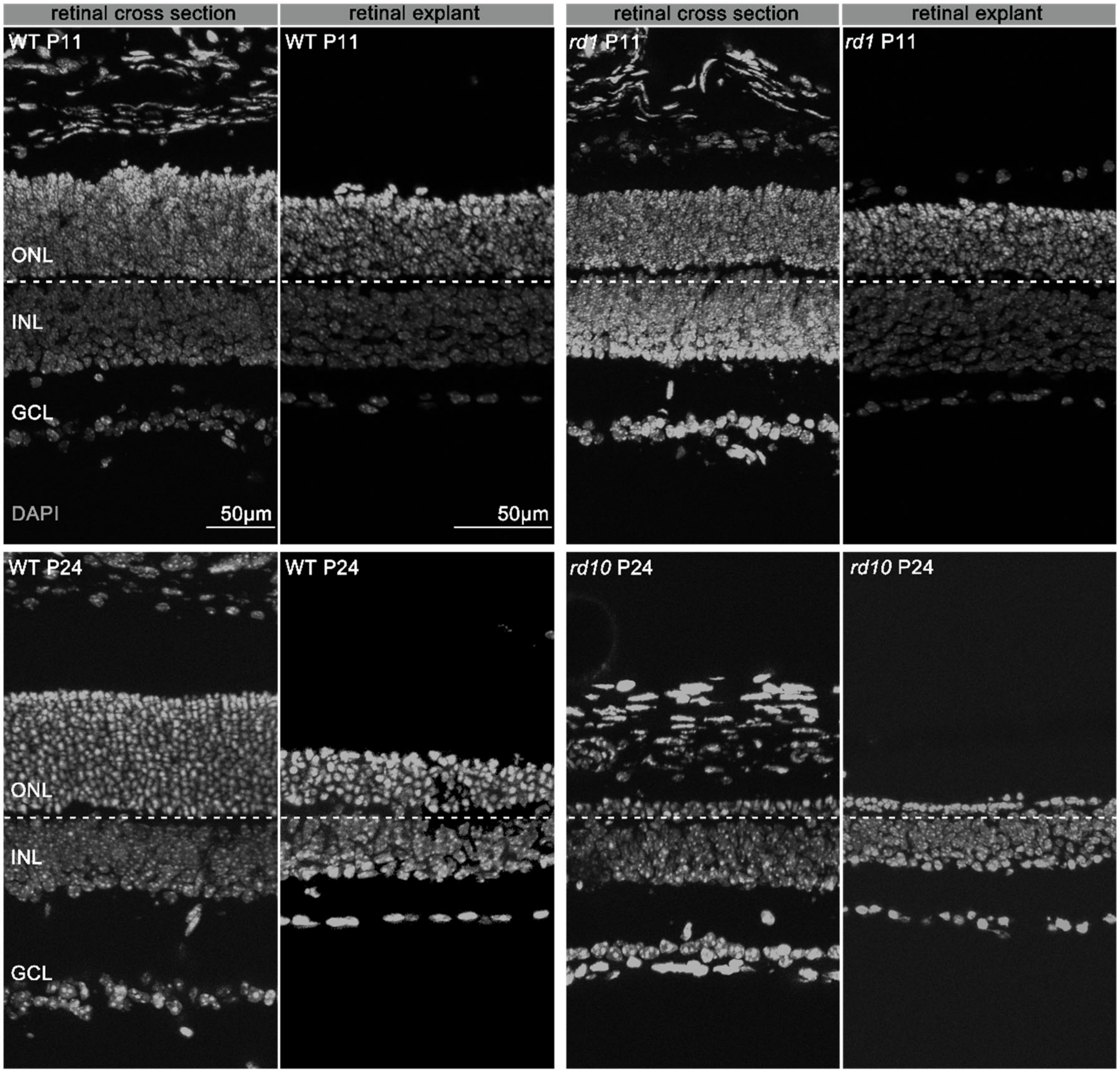

Figure S7. Comparison of wild-type, *rd1*, *rd10* retina; *in vivo vs. in vitro*. Retinal cross-sections were stained with DAPI (grey) to label cell nuclei. Top row: Cross-sections obtained at post-natal (P) day 11 from either wild-type (WT) or *rd1* retina. The *in vivo* situation is shown on left while the situation after six days of *in vitro* culture is displayed on right. Bottom row: WT and *rd10* retina at P24 *in vivo* (left) and *in vitro*, after 12 days of culture (right). Note that while the *in vitro* retina appears somewhat thinner than its *in vivo* counterpart, it maintains the general tissue architecture and cell viability even after 12 days of culture. ONL = outer nuclear layer, INL = inner nuclear layer, GCL = ganglion cell layer.

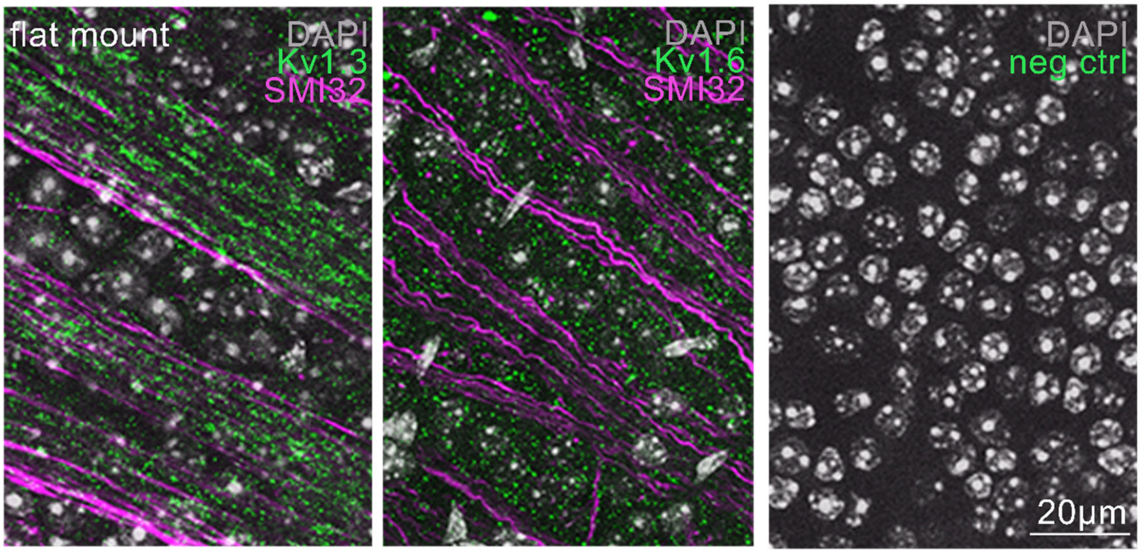

Figure S8. Localization of Kv1.3 and Kv1.6 in mouse retina. Flat-mounted retinae derived from post-natal day 24 (P24) wild-type mice stained with DAPI (grey), for Kv1.3, Kv1.6 (both green), and the axonal marker SMI32 (magenta).

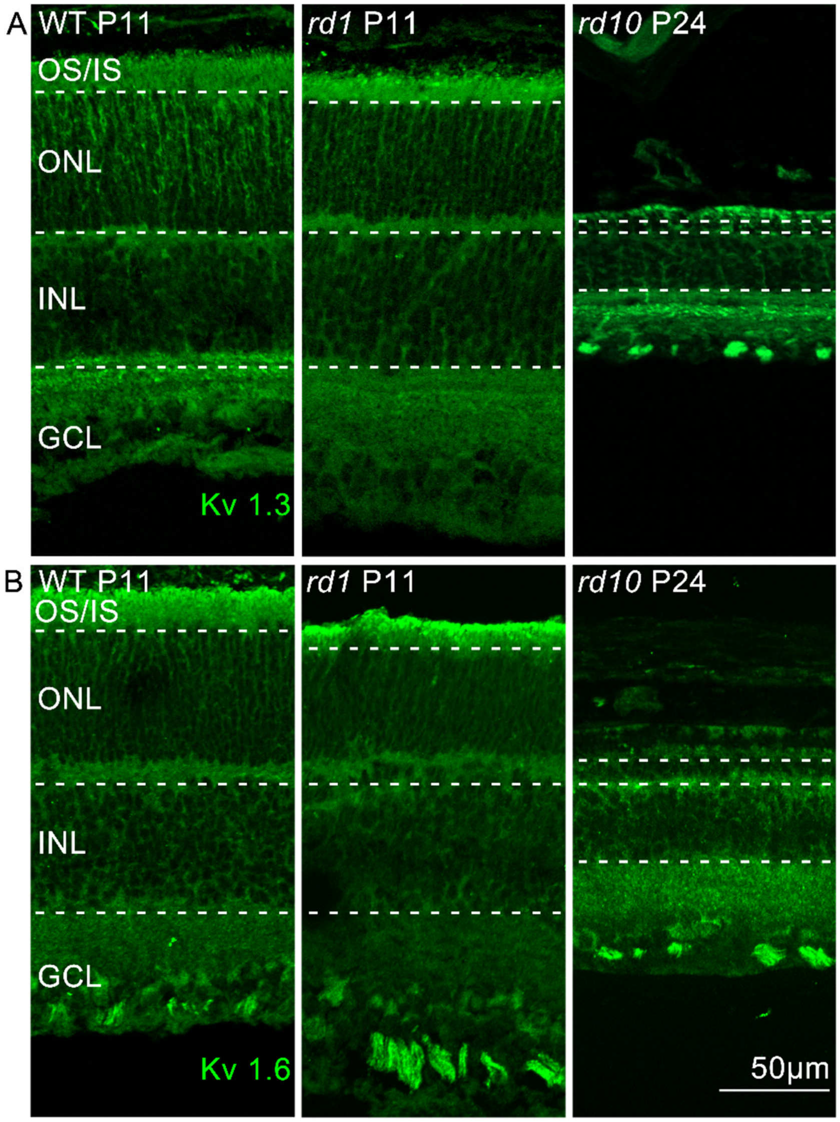

Figure S9. Comparison of Kv1.3 and Kv1.6 localization in wild-type, *rd1*, *rd10* retina; Retinal cross-sections derived from wild-type (WT) and *rd1* retina at post-natal (P) day 11 and from *rd10* retina at P24 were stained for Kv1.3 and Kv1.6 (both green). OS = outer segment, IS = inner segment, ONL = outer nuclear layer, INL = inner nuclear layer, GCL = ganglion cell layer.

Table S1. **Statistical analysis of the effect of different cGMP analogues on Kv1.3 channel activity.** The indirect effect of PKG -activators and -inhibitors was measured after 2-, 4-, 6-, 8-, 10-, and 15-minutes following cGMP-analogue application. The respective values were compared with Kv1.3 channel activity measured at the same time intervals, in the absence of the respective analogues.

**cGMP analogue**		**2 min.**	**4 min.**	**6 min.**	**8 min.**	**10 min.**	**15 min.**
	**N**	***p*-value**	***p*-value**	***p*-value**	***p*-value**	***p*-value**	***p*-value**
**CN008 (8-Br-PET-cGMP)**	4	≥ 0.05 (*ns*)	≥ 0.05 (*ns*)	≥ 0.05 (*ns*)	≥ 0.05 (*ns*)	≥ 0.05 (*ns*)	≥ 0.05 (*ns*)
**CN052 (8-Br-pMe-PET-cGMP)**	5	≥ 0.05 (*ns*)	≥ 0.05 (*ns*)	≥ 0.05 (*ns*)	≥ 0.05 (*ns*)	≥ 0.05 (*ns*)	≥ 0.05 (*ns*)
**CN003 (Rp-8-Br-PET-cGMPS)**	5	0.0235 (*)	0.0033 (**)	0.0052 (**)	0.0072 (**)	0.0005 (***)	0.0001 (***)
**CN238 (Rp-8-Br-pMe-PET-cGMPS)**	5	≥ 0.05 (*ns*)	≥ 0.05 (*ns*)	≥ 0.05 (*ns*)	≥ 0.05 (*ns*)	0,0375 (*)	0,0338 (*)

Table S2. Fluorophores and microscope filters. Excitation (exc.) and emission (em.) characteristics of the TMR red, AF488 and AF568 and of the microscope filter sets used for their visualization.

**Fluorophore**	**exc. max. / em. max. (nm)**	**exc. filter / em. filter (nm)**
**TMR red**	540/580	538-562/570-640
**AF488**	495/519	450–490 /500-550
**AF568**	577/603	538-562/570-640
